# Estrogen Receptor Signaling in Breast Cancer

**DOI:** 10.3390/cancers15194689

**Published:** 2023-09-23

**Authors:** Paulina Miziak, Marzena Baran, Ewa Błaszczak, Alicja Przybyszewska-Podstawka, Joanna Kałafut, Jolanta Smok-Kalwat, Magdalena Dmoszyńska-Graniczka, Michał Kiełbus, Andrzej Stepulak

**Affiliations:** 1 Department of Biochemistry and Molecular Biology, Medical University of Lublin, 1 Chodzki Street, 20-093 Lublin, Poland; marzenabaran@umlub.pl (M.B.); ewa.blaszczak1@umlub.pl (E.B.); alicja.przybyszewska-podstawka@umlub.pl (A.P.-P.); joannakalafut@umlub.pl (J.K.); magdalena.dmoszynska-graniczka@umlub.pl (M.D.-G.);; 2 Department of Clinical Oncology, Holy Cross Cancer Centre, 3 Artwinskiego Street, 25-734 Kielce, Poland; jolantasm@onkol.kielce.pl

**Keywords:** estrogen receptor, ER, breast cancer, ER signaling, ER coregulators, post-translational modifications, therapeutic targeting

## Abstract

**Simple Summary:**

Estrogens, belonging to a group of steroid compounds, play an important role in both physiological and disease processes, mainly by interacting with estrogen receptors (ERs). Abnormal ER signaling may result in various cancers, including breast cancer (BC), one of the most often diagnosed cancers in women globally, and a second cause of female cancer-related death. In the present review, we discuss the current knowledge of the estrogen receptor-dependent signaling pathways in breast cancer. The significance of clinical implications of ER signaling in BC, including the potential therapies, is also summarized.

**Abstract:**

Estrogen receptor (ER) signaling is a critical regulator of cell proliferation, differentiation, and survival in breast cancer (BC) and other hormone-sensitive cancers. In this review, we explore the mechanism of ER-dependent downstream signaling in BC and the role of estrogens as growth factors necessary for cancer invasion and dissemination. The significance of the clinical implications of ER signaling in BC, including the potential of endocrine therapies that target estrogens’ synthesis and ER-dependent signal transmission, such as aromatase inhibitors or selective estrogen receptor modulators, is discussed. As a consequence, the challenges associated with the resistance to these therapies resulting from acquired ER mutations and potential strategies to overcome them are the critical point for the new treatment strategies’ development.

## 1. Introduction

One of the most often diagnosed malignancies in women globally is breast cancer (BC), being now the second cause of death because of cancer [1,2]. The biological activity and treatment response of BC are influenced by a variety of histological and molecular abnormalities [3]. Despite improvements in the development of diagnostic methods and treatments, the incidence and mortality rate of breast cancer-bearing patients are rising internationally [4]. Age, family history, histological differentiation and grading, and the local and systemic advancement of the disease have all been studied to evaluate the patient risk and choose the best course of action [5,6]. The three main types of breast cancer are classified based on the hormone receptors’ status. The first group consists of tumors that have either tested positive for the estrogen receptor (ER) or the progesterone receptor (PR). The second group consists of tumors that have either tested positive for the human epidermal growth factor receptor 2 (HER2) with or without ER and PR positivity, whereas the third one is called triple-negative breast cancer (TNBC), since these types of tumors lack expression of all three receptors (ER, PR, HER2) [7]. Receptor status, among other variables, has been demonstrated as the one of most important factors in estimating the prognosis and therapeutic response [8]. Furthermore, breast cancer classification based on intrinsic molecular subtypes as a result of the microarray expression profiling has been distinguished [9,10]. These are termed luminal A (ER+PR+ tumors, expressing luminal genes such as *ESR1*, *GATA3*, *XBP1*, and *FOXA1*; characterized by the low expression of Ki-67), luminal B (ER+ with lower expression of luminal genes, e.g., *PGR* and *FOX1* and a high expression of Ki-67, >20%), HER2-enriched (characterized by the HER2 positivity; however, not all clinically classified HER+ tumors are of these molecular subtype and intermediate expression of luminal genes), basal-like (increased expression of *EGFR* and basal cytokeratins with low expression of the luminal A-type genes), and claudin-low (ER-, PR-, and HER- tumors are also negative for claudin 3/4/7 and E-cadherin (reviewed in: [11,12,13]).

ERs are activated by estrogens and play important roles in the development of several cancers; in particular, breast [14], endometrial [15], and ovarian cancers [16]. Estrogens are a group of low molecular weight lipophilic molecules that occur in three forms: estrone (E1), estradiol (E2; the term estrogen is used in relation to E2, due to its predominant role in physiology), and estriol (E3) [17]; the fourth form produced during pregnancy, namely estetrol (E4), is a fetal estrogen with selective tissue actions [18]. These hormones contain in their structure a steroid skeleton made of four aromatic rings. One of them is the phenolic A ring, which is responsible for binding to the ER [19]. Estrogens, like other steroid hormones, are synthesized at the rough endoplasmic reticulum from its precursor—cholesterol, which is described in detail by Fuentes and Silvera (2019) [20]. Briefly, they are synthesized from androstenedione in the presence of oxygen and NADPH. The crucial enzyme involved in this process is aromatase (CYP19A1), an enzyme that participates in the final stage of E1 and E2 synthesis. The synthesis of estrogens takes place in the gonads (predominantly in the ovaries—granulosa cells), adrenal cortex, and adipose tissue, in smaller amounts also in other tissues, including breast and placenta [21], or fetal liver, in the case of E4 [18]. E1 and E2 can arise from testosterone in peripheral tissues (mainly adipose tissue) in the enzymatic reaction catalyzed via aromatase, which has a significant impact on the level of estrogen synthesis in postmenopausal women [22].

Estrogens, including E2(the predominant circulating estrogen in humans) are transported in the blood along with specific proteins. They sequentially cross biological membranes by diffusing to the target sites, where they primarily act by attaching to specific nuclear ER. Receptor–ligand complexes can directly silence/activate gene expression or act indirectly by interacting with intracellular signaling molecules. The mechanism of action of estrogens is very diverse, and the nature of the response depends on both the genetic and physiological predisposition of the target cells. Estrogens are synthesized in both sexes; however, at different concentrations and with different functions [23]. These hormones play a significant role in the proliferation and growth of cells associated with reproduction and have a myriad of other cellular functions; for instance, carbohydrate and lipid metabolism, and the regulation of energy homeostasis [17,24]. Importantly, estrogens affect the cardiovascular [25] and central nervous system [26]. The effect of estrogens on the cardiovascular system may be protective, as shown by several studies, including large-scale clinical trials [27,28,29], but have also been associated with the risk of coronary heart disease [30]. Furthermore, estrogen-related malfunctions result in several autoimmune, metabolic, or degenerative pathologies and cancers, including the development of breast cancer [17].

The ER plays a key role in the development, progression, and invasion of ER-expressing BC [31]. ER-positive tumors have a more favorable prognosis compared to other BC types and are usually responsive to hormonal treatment. In the absence of ERα expression, BC exhibits more aggressive phenotypes [32]. Here, we discuss the current knowledge of the ER-dependent signaling in breast cancer. The review highlights the molecular traits of estrogen receptors and presents the ERs’ coregulators. Post-translational regulation via various modifications of ER is also presented. Finally, the challenges related to the current therapies and the potential strategies to overcome them are summarized.

## 2. Estrogen Receptors

The ER family includes the nuclear ER (nER) and G protein-coupled estrogen receptor 1 (GPER1) [33]. nER is characterized by conserved domain structures, such as the DNA-binding domain (DBD) and the ligand-binding domain (LBD) [34]. Two major nER isoforms, ERα and Erβ, are responsible for the regulation of the female reproductive system development, the preservation of bone mass, and the protection of the central nervous system, among other physiologically important processes [35]. The evolutionary origin of the estrogen-signaling system remains unclear; however, the research on invertebrates provided insight into the vertebrate pathway. Interestingly, the ER homologs have been identified in amphioxus [36,37], mollusks [38,39], and annelids [40]. Regarding the functional insights, the ERs from amphioxus and mollusks are not activated by estrogens [38,41,42], while in two annelid species, transcription is activated in response to the low doses of estrogens upon ER binding [40]. Based on the phylogenetic context, it was hypothesized the ER possibly originated in the bilateralian lineage [43]. In humans, the nERs are encoded by two different genes (*ESR1* for ERα [44] and *ESR2* for ERβ [45]) as a result of gene duplication in the early vertebrate lineage [46] that are located on different chromosomes—*ESR1* is located on chromosome 6 and *ESR2* on chromosome 14.

**Figure 1:**
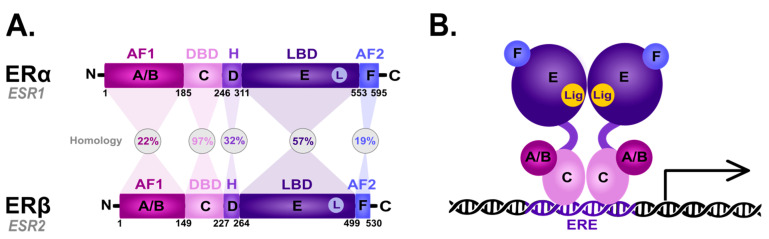
Scheme of the structural and functional regions of the estrogen receptor (ER). (**A**) Comparison of the domain topology of ERα and ERβ. The homology of the ERα and ERβ receptors was determined based on the amino acid sequence retrieved from the UniProt database (https://www.uniprot.org/; accessed on 12 March 2023; ESR1 ID: P03372, ESR2 ID: Q92731). AF1—the activation of function domain 1; DBD—the DNA-binding domain; H—hinge region; LBD—ligand-binding domain; AF2—the activation of function domain 2; A/B—the domains located at the N-terminus (N); C—the domain containing zinc-finger; D—the domain with nuclear localization signal; E/F—the domains located at the C-terminus (C). (**B**) Diagram of the estrogen receptor dimer binding to DNA in the estrogen-response elements (ERE). A-F as explained in the description of A; Lig—ligand.

The nER is composed of six homologous A-F domains (Figure 1) representing the receptors’ structural regions and having unique functional characteristics. Domains A and B are located at the amino terminus (N-terminal domain) and contain the so-called activation of function domain 1 (AF-1), whose function is to activate the transcription of target genes [20]. Domain C possesses a zinc-finger motif and corresponds to the DBD domain, namely the DNA-binding domain. This domain is responsible for receptor dimerization and binding to the estrogen-dependent genes promoters’ sequences, called estrogen-response elements (ERE) [47]. The D domain is characterized by the presence of a nuclear localization signal (NLS), which, after the binding of a specific ligand, followed by the conformational change caused by this interaction, is exposed, and it is necessary for translocation to the nucleus. Domain D is the so-called hinge region (H), which is responsible for the functional synergy between fragments AF-1 and the second transcriptional activation domain—the AF-2 fragment located at the carboxyl terminus (C-terminus) [48]. The E domain is the ligand-binding domain (LBD), which contains the ligand-binding site (L). The F domain located at the end of the C-terminus probably acts as a modulator of transcriptional activity and is involved in the interaction with the coactivators [49,50].

ERα and ERβ show high homology in the LBD and DBDs, while they differ in the transcription-activating domain (AF-1) [20]. Due to alternative splicing, both receptor subtypes occur in isoforms [20,51,52,53,54]; five shorter isoforms for ERα, and three shorter isoforms and one longer isoform for ERβ [20]. They are also differentially expressed throughout the body [55,56]: ERα predominance is shown by the endometrial cells, ovary, hypothalamus. and outgoing ducts’ testicles, while ERβ is expressed mainly in the kidney cells, brain, heart, bones, lungs, intestinal mucosa, prostate, and vascular endothelium. The deregulation of ERα expression and function is closely related to the carcinogenesis process in ovarian, uterine, and breast cancer epithelial cells. On the other hand, ERβ inhibits ERα-mediated transcription and estradiol-induced cell proliferation, which is probably the reason why it is associated with benign forms of breast cancer [57,58,59]. The ERα/ERβ cellular ratio plays a key role in regulating E2 activity; for instance, in human T47D BC cells [60]. However, approximately 75% of breast tumors are ER-positive [61] and aberrations in the function are associated with ERα. Hence, ERα is one of the main clinical drug targets [62]. The primary function of both receptors is the downstream regulation of gene transcription upon E2 binding to control the cell proliferation and differentiation activated by the ER-dependent signal transduction [63].

GPER1 (also known as GPR30), is the second type of estrogen-dependent receptor and is a member of the transmembrane metabotropic receptors family, which was originally detected in breast cancer tissue [64]. The GPER1 coding gene is located on chromosome 7 [65]. It is created via a single polypeptide with an α-helical structure strongly folded and immersed in the cell membrane, through which the polypeptide chain passes seven times, forming a hydrophobic transmembrane domain [66]. The GPER1 is present in many cells and tissues. mRNA expression was confirmed, e.g., in the ovaries, prostate, thymus, bone marrow, skeletal muscles, liver, lungs, heart, kidney, pancreas, small intestine, and brain [67]. In response to the extracellular signal by its predominant ligand—E2, the GPER1 regulates many cellular processes via a rapid non-genomic dependent mechanism. Compared to normal tissues, GPER1 is detected with a higher expression in breast cancer cells [68].

## 3. Estrogen Signaling

### 3.1. Genomic Action of ER

**Figure 2:**
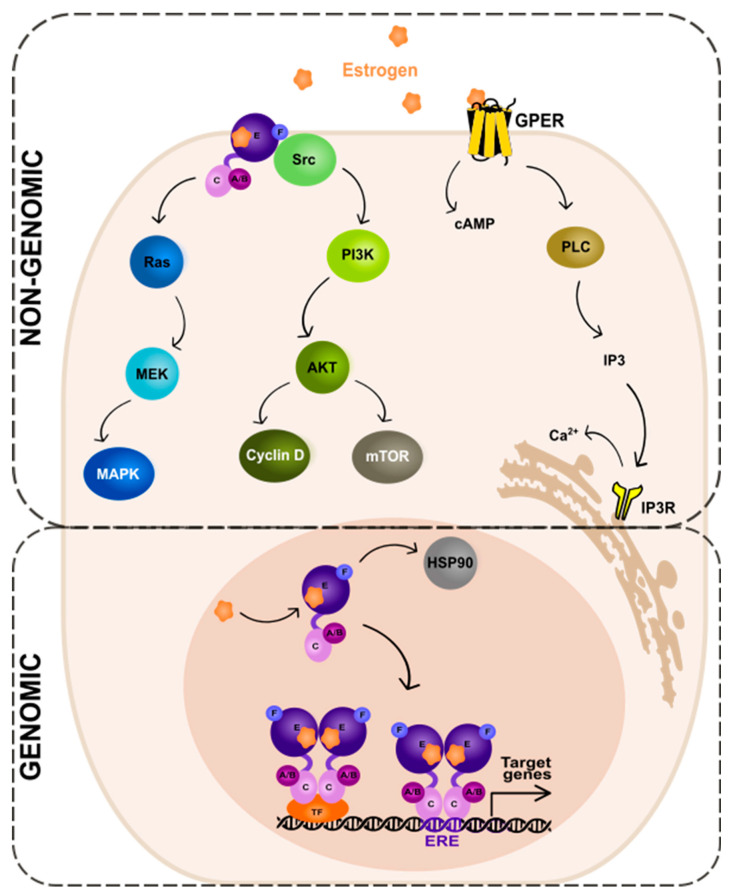
Genomic and non-genomic action of estrogen receptor (ER). Abbreviations: A/B—the domains of ER located at the N-terminus of estrogen receptor (N); C—the domain containing zinc-finger; E/F—the domains located at the C-terminus; GPER—G protein-coupled estrogen receptor 1; PI3K—phosphatidylinositide 3-kinase; AKT—serine/threonine kinase; mTOR—the mammalian target of rapamycin; cAMP—cyclic adenosine monophosphate; PLC—phospholipase C; IP3—inositol trisphosphate; IP3R—inositol trisphosphate receptor; HSP90—heat shock protein 90; ERE—estrogen-response element; TF—transcription factor.

The ER-dependent signaling can be classified as genomic and non-genomic with different activities and pathways involved, respectively (Figure 2). Genomic signaling (Figure 2; bottom panel) depends on the transcriptional activities via the gene expression, while non-genomic (Figure 2; top panel) depends on the activation of various signaling cascades, as reviewed in: [20,69].

In the genomic ER signaling, the complexes of estrogen and the estrogen receptor (ER) are translocated to the nucleus. There, they can indirectly bind to the DNA-binding transcription factors (TFs) via the TF response elements, using protein–protein interactions. By interactions with the coactivator proteins, ER can control the activation of TFs [70]. Nuclear ER can, for example, interact with specificity protein 1 (Sp1) and nuclear factor kappa B (NF-κB) via the so-called “non-classical” activity [71]. The target genes to be modified by the indirect action of ER do not contain the estrogen-response elements (EREs) in their promoters’ regions.

The expression of genes that contain EREs can be changed via the direct genomic action of ER. The receptor undergoes a ligand-specific conformational shift after ligand attachment to the ER, enabling the receptor to be released from the heat shock protein complex (HSP90) [72,73]. HSP90 is a molecular chaperone, which protects unbound ER from degradation [74]. Eckert and colleagues have shown nearly 40 years ago [75] that ERα without a ligand is a constantly degraded, short-lived protein (a half-life of 4–5 h). The ERα synthesis and turnover rates were determined in the MCF-7 breast cancer cells. For complete ER-mediated transcriptional activation, histone acetyltransferases (HATs) are necessary. HATs activities enable nucleosome repositioning, chromatin opening, and engagement with the general transcription machinery centered on RNA polymerase II. For example, the p300/CBP acetylates elements of the basal transcription machinery and interacts with other HATs, such as PCAF [76,77,78].

Importantly, there is functional crosstalk between the estrogen receptor and other steroid hormone receptors, such as the progesterone receptor (PR), glucocorticoid receptor (GR), and androgen receptor (AR) in breast cancer cells [79,80,81,82,83,84,85], as well as other cancer cell types, like endometrial [86,87]. These hormones have similar DNA-binding preferences and their genomic binding orchestrates the recruitment of other TFs and chromatin remodeling complexes [88,89,90]. Clearly, ER does not function on its own, and its action can be altered by other receptors. For instance, while co-expressed in BC cells, PR is not only an ERα-induced target gene but also an ERα-associated protein, which redirects ERα-associated chromatin binding events [81,84]. This, in turn, results in a unique gene expression in BC cells and is associated with patients’ outcome [81]; however, the mechanistic insight into ER modulation via PR for better BC management needs to be elucidated [84]. AR has also been shown to play a role in ER genomic binding in breast cancer [82] and its function and targeted therapies across BC subtypes have recently been reviewed in [91]. Additionally, in breast cancer cells, the liganded glucocorticoid receptor represses an ERα-regulated transcriptional program [92]. Tonsing-Carter and colleagues [93] have shown that GR modulation decreases ER-positive BC cells’ proliferation and suppresses ER (both wild-type and mutant) chromatin association.

### 3.2. Non-Genomic Action of ER

In the non-genomic ER signaling (Figure 2; top panel), estrogen binds to the receptor (mbER, i.e., the ER that is situated at the plasma membrane [94] or GPER1, the G-protein-coupled estrogen receptor 1 [95]). This mechanism starts outside of the nucleus and is unrelated to the transcription. The estrogen and ER complexes predominantly activate the kinase pathways. These include MAPK (mitogen-activated protein kinase) via the so-called Ras-Raf-MEK-MAPK pathway and PI3K (phosphatidylinositide 3-kinase)/AKT (serine/threonine kinase) via the PI3K-AKT-mammalian target of rapamycin (mTOR) pathway. The activation of the MAPK signaling pathway by estrogen has been studied in various cell types, including breast cancer [96], neuroblastoma [97], and endothelial [98] cells. Upon estrogen binding to the receptor, the small guanine nucleotide-binding protein—Ras (GTPase) is activated. Next, another protein kinase—Raf is activated, which then phosphorylates the MEK protein. This in turn leads to the phosphorylation and activation of MAPK. As a consequence, several TFs of the activating protein 1 family, e.g., c-Jun and c-Fos, are activated. These then regulate the transcription of the target genes [99,100,101].

An alternate pathway—the PI3K-AKT-mTOR, activated by mbER, relies on the direct contact of ER with different proteins; first, the tyrosine kinase Src, then the phosphatidylinositol 3-kinase (PI3K), and the AKT proteins that regulate the mTOR pathway. The AKT-dependent mechanisms of mTOR regulation is a key intracellular system that signals cellular growth and survival, and the hyperactivation of it is involved in the carcinogenesis of the ER-positive BC as well as the resistance to endocrine therapy [102].

The activation of receptors connected to G-proteins is another well-known non-genomic effect of sex hormones. GPER1 is a transmembrane receptor, which, once activated by estrogen or its derivatives, triggers the downstream signaling pathways that can affect a variety of physiological processes [95], such as cell proliferation, angiogenesis, and inflammation. The action of GPER1 generates cyclic adenosine monophosphate from the activation of the adenylate cyclase enzyme. Moreover, upon activation of a receptor by estrogen, the PLC (phospholipase C) enzyme is activated. The activated PLC cleaves phosphatidylinositol 4,5-bisphosphate (PIP2) into two secondary messengers, inositol 1,4,5-trisphosphate (IP3) and diacylglycerol (DAG). IP3 diffuses into the cytoplasm and binds to the IP3 receptors on the endoplasmic reticulum, leading to the release of Ca^2+^ from the endoplasmic reticulum into the cytoplasm. This results in a rapid increase in intracellular Ca^2+^ concentration, which can trigger a variety of downstream signaling events. DAG, on the other hand, remains in the plasma membrane and activates protein kinase C (PKC), another downstream signaling molecule that can regulate various cellular processes [103].

## 4. Coactivators and Corepressors of ER in Breast Cancer

The activity of estrogen receptors is closely coordinated by its coregulators—coactivators and corepressors. The coregulators’ complexes are recruited by the ER via a characteristic, conserved LxxLL motif [104] (L, leucine; x, any amino acid) that acts on the LBD. Coregulators are often associated with enzymes such as methyltransferases, acetylases/deacetylases, phosphokinases, ubiquitin ligases, and ATPases. They regulate chromatin remodeling, and thus, gene expression [78,105]. The interaction between coactivators and corepressors shapes the transcriptional landscape, which is cell type- and context-dependent. Corepressors interact with histone deacetylases (HDAC) by attaching to the target chromatin of the ERα-encoding gene. This results in chromatin condensation and the inhibition of ERα gene expression. Corepressors are designated to balance the activity of coactivators and inhibit the excessive expression of the nuclear receptor-encoding gene [105]. Aberrations in the nuclear receptor coregulators’ expression or activity is closely related to carcinogenesis, tumor invasion, and metastasis, as observed in breast, colorectal, and other cancer types [106]. The main nER coregulators, including coactivators and corepressors, are described in Table 1.

## 5. Post-Translational Modifications of Estrogen Receptors

ERs undergo various post-translational modifications (PTMs) that regulate their activity and contribute to the development and progression of breast cancer. The PTMs of ERs are versatile with a large spectrum of functional diversity influencing the subcellular localization of ERs, with their expression and stability or sensitivity to the hormonal response [73,141]. The modifications include phosphorylation, acetylation, methylation, ubiquitylation, sumoylation, and several more, with usually many sites distributed over the ER. Some PTMs of estrogen receptors, including those occurring particularly in breast tumors, are listed in Table 2.

Phosphorylation, first described as the PTM of ER, can activate or repress ER function, depending on, e.g., the site of modification [142]. For instance, the phosphorylation of serine 118 (S118; the most-studied modification) in ERα by MAPK kinases enhances its transcriptional activity, while the phosphorylation of serine 167 (S167) in ERα by AKT inhibits its DNA binding and transcriptional activity [143,144]. Chromatin immunoprecipitation experiments have shown that S118 phosphorylation localizes to several promoters of target genes, which demonstrates its role in transcription [145]. The other phosphorylated serines may also regulate the properties of ERα. For example, serine 236 (S236), phosphorylated by PKA (protein kinase A), plays a role in receptor dimerization [146]. The phosphorylation of threonine 311 (T311) by p38-MAPK inhibits the nuclear export of ERα [147]. Other studies have shown that calcineurin, a Ca^2+^-dependent serine/threonine phosphatase, stabilizes and activates ER. This is due to the suppressive effects of PP2A and PP5 (serine/threonine protein phosphatases) on the ER. The ubiquitin ligase E6AP was stimulated to the polyubiquitylate-phosphorylated estrogen receptor (ER S294) and, as a consequence, led to its proteasomal degradation. By directly dephosphorylating ER S294 and releasing E6AP, calcineurin stabilized the ER. Moreover, calcineurin could lead to the phosphorylation of ER S118 via activating the Akt-mTOR pathway. In patients with ER-positive breast cancer receiving endocrine therapy, higher calcineurin expression was linked to shorter recurrence-free survival [148]. Importantly, multifunctional enzymes with a primary role in phosphorylation, namely CDK4/6 (cyclin-dependent kinases 4 and 6) have been shown to improve the progression-free (PF) and overall survival (OS) of ER-positive breast cancer patients [149]. However, the success of these treatments is still limited due to the acquired patients’ resistance to these inhibitors [150].

ERs can also be acetylated by histone acetyltransferases (HATs) such as p300/CBP and PCAF. The acetylation of ERα at lysine 302 and 303 (K302 and K303) enhances its DNA binding and transcriptional activity. The acetylation in the hinge (H) domain of ERα through its coregulatory protein p300 but not PCAF, changes the ligand sensitivity and causes a subsequent histone deacetylation effect [151]. p300 also acetylates K266 and K268, stimulating the binding of the receptor to DNA and consequently enhancing its transcriptional activity [152]. Interestingly, the acetylation of a lysine residue in histone protein H3 at position 27 (H3K27) has recently been shown to signal transcriptional elongation for ERα. The so-called super elongation complex (SEC) interacts with an acetylated H3 on the *ESR1* transcription start site (TSS) via the scaffold protein AFF4. This protein functions as a key molecule in the transcriptional elongation machinery [153]. Enhanced ER coregulator’s interactions via an acetylation-dependent activation of ERα have potential implications in breast cancers, e.g., *SRC-3*/*AIB1* gene amplifications and ERα gain-of-function mutations in endocrine-resistant metastatic tumors, such as Y537S and D538G [154]. It has recently been reported that the pharmacological inhibition of p300/CBP HATs through inhibitors A-485 and GNE-049 downregulates ERα via suppressing H3K27 acetylation in ER-positive breast cancer [155].

Methylation is another modification that controls ER gene transcription and correlates with resistance to hormone therapy [156]. The promoters of genes implicated in particular biochemical pathways are methylated in a number of cancers, including breast cancer (reviewed in: [157]). Several studies reported differential promoter methylation statuses for ER/PR-negative versus ER/PR-positive tumors [158,159,160,161,162]. Therefore, DNA methylation markers have the potential to provide predictive value in the treatment of breast cancer. While lysine (K) methylation is often associated with histones, non-histone proteins, such as p53 or DNA methyltransferases, have been shown to undergo methylation. Lysine can be mono-, di-, or trimethylated [163]. For example, the SET7 lysine methyltransferase catalyzes the monomethylation of ERα at K302, which affects ERα stability. This modification thus facilitates the ERα binding to target genes for transactivation [164].

Another PTM that may affect the ER is ubiquitylation. This process relies on a small, highly conserved protein ubiquitin (Ub) to be attached to the K residues of a substrate protein (reviewed in: [165]), having either proteolytic (proteasomal degradation) [166] or non-proteolytic fates for the target substrate [167,168]. The ubiquitylation process is a complex mechanism with several structurally-related enzymes involved, namely ubiquitin-activating enzymes, ubiquitin-conjugating enzymes and ubiquitin ligases, and Ub itself having the ability to form ubiquitin chains with different linkages [169]. The aberrations in any stage of this process are associated with various pathologies, including breast cancer [170] and many other cancer types. It was not until 2008 that two ERα lysines, located at the H region of the receptor, namely K302 and K303, were identified as ubiquitylated, regulating the stability of ERα. The polyubiquitylation of ERα on these lysines plays a role in activating the transcriptional activity of estrogen-dependent ERα. The receptor degradation is regulated by the proteasome [171]. Several ubiquitin ligases and deubiquitylating enzymes (DUBs; as the process is reversible) are associated with the control of ER transcriptional activity and stability [172,173]. Tang and colleagues [174] described the novel TRIM11 ubiquitin ligase function in ER signaling. The level of TRIM11 highly correlates with ERα and the depletion of this ligase in BC cells decreases cell proliferation and migration. The ERα stability is increased via monoubiquitylation [174]. Moreover, studies by Xiao and colleagues [175] have shown that the inhibition of transcriptional repressor, ZBTB7A, which promotes the progression of breast cancer, can upregulate E3 ligase TRIM25. As a consequence, this leads to an increased level of ERα ubiquitylation and its proteasomal degradation [175]. Importantly, a new therapy targeting ER degradation, such as proteolysis-targeting chimeric (PROTAC) technology, is being developed. It targets the regulation of ER stability via ubiquitylation, a therapeutic target for breast cancer [176,177].

A post-translational modification called sumoylation controls the activity and localization of the ER. Similarly, as for ubiquitylation, the term sumoylation refers to the covalent attachment of a tiny protein known as the small ubiquitin-like modifier (SUMO) to the substrate protein (reviewed in: [178]). The sumoylation of the ER has generally been proven to increase its nuclear localization and suppress its transcriptional activity [179]. Although ERα lacks consensus sumoylation sites, Sentis and colleagues have shown that the receptor was sumoylated in the H region on K266, K268, K299, K302 and K303. The sumoylation of ERα is estrogen-dependent and involves the sumo ligase PIAS1 (protein inhibitor of activated STAT1) and PIAS3 (protein inhibitor of activated STAT3). The sumoylation of ERα increases its transcriptional activity [179]. Recent studies have also shown that several antiestrogens (estrogen blockers/inhibitors), used to treat ER-positive breast cancer induce the sumoylation of ERα, but not ERβ [180].

**Table 2:** The selected post-translational modifications of ERs.

Site of Modification	Type of Modification	Enzymes	Functions	Reference
Y52	phosphorylation	c-Abl	transcription activation, stability maintenance	[181]
Y219	phosphorylation	c-Abl	DNA binding and dimerization	[181]
S102	phosphorylation	GSK3	transcription activation	[182]
S104/106	phosphorylation	cyclin A-Cdk2, MAPK	transcription activation, dimerization	[182]
S118	phosphorylation	ND, Cdk7, IKKα	RNA splicing, dimerization, transcription activation	[182,183]
S167	phosphorylation	Akt, p90 RSK, S6K1	stability maintenance	[184]
S236	phosphorylation	PKA	dimerization inhibition	[146]
R260	methylation	PRMT1	non-genomic signaling	[185]
K266	acetylation	p300	transcription activation, DNA binding	[146]
K266 K268	sumoylation	Ubc9, PIAS1, PIAS3	transcription activation, DNA binding	[179]
S282 S559	phosphorylation	CK2	transcription inhibition	[186]
K302 K303	ubiquitylation	CHIP	proteasomal degradation	[171]
K302	acetylation	p300	transcription inhibition	[151]
K302	methylation	SET7	regulation of ER turnover	[164]
K303	acetylation	p300	transcription inhibition	[151]
K303	sumoylation	Ubc9, PIAS1, PIAS3	transcription activation, DNA binding	[179]
S305	phosphorylation	PAK1	resistance to aromatase inhibitor, transcription activation	[187,188]
T311	phosphorylation	p38-MAPK	nuclear localization	[147]
C447	palmitoylation	PAT	plasma membrane localization	[189,190]
Y537	phosphorylation	calf uterine kinase, SRC, EGFR	DNA binding, dimerization, proliferation	[191,192]

## 6. Estrogen Receptor Mutations

**Figure 3:**
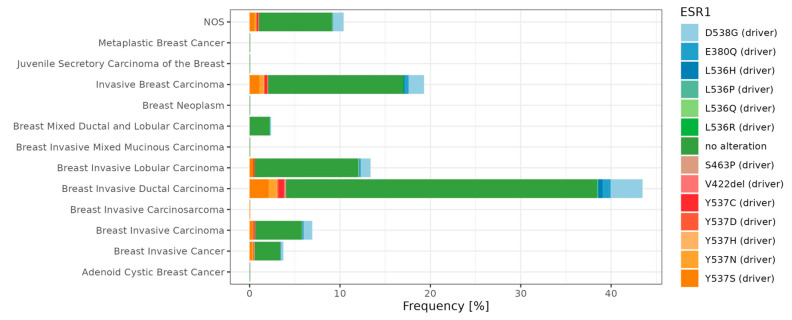
Frequencies of *ESR1* single nucleotide variants among breast cancer subtypes. The percentage of counts is presented based on Metastatic Breast Cancer (MSK, Cancer Discovery 2022, *n* = 1116) repository. The dataset was obtained from the cBioPortal database [197] (https://www.cbioportal.org/; accessed on 18 September 2023) and visualized in R software (R version 4.1.3, ggplot2 R package version 3.4.3).

**Figure 4:**
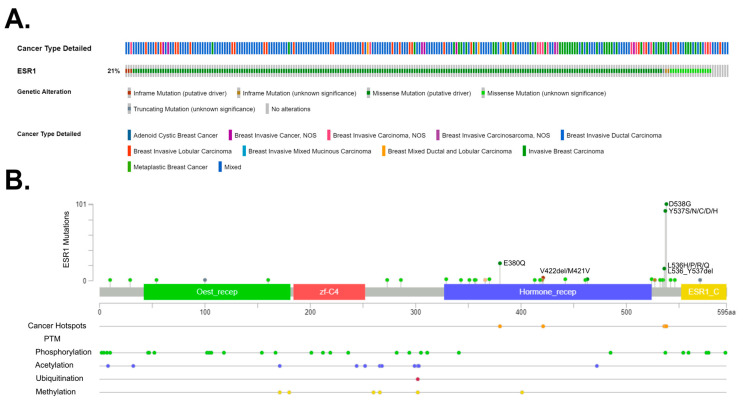
Frequency and location of mutations in the estrogen receptor 1 (*ESR1*) gene. (**A**) *ESR1* gene mutations, found in 21% of 1116 breast cancer patients, based on the Metastatic Breast Cancer (MSK, Cancer Discovery 2022) repository with the mutation types. (**B**) The distribution of mutations in the *ESR1* gene indicates the presence of three cancer hotspots. The main post-translational modifications were also indicated. All data were obtained via the cBioPortal database [197] (https://www.cbioportal.org/; accessed on 15 March 2023).

Overall, many genes bearing various mutations have been identified as involved in tumorigenesis. These so-called mutational cancer diver genes have been reviewed in detail by Martínez-Jiménez and colleagues [193]. In breast cancer, Krøigård and colleagues identified metastasis driver genes [194], and the study of Zhang and colleagues [195] characterized the frequency of mutation in Chinese BC patients. Recently, Nolan and colleagues [13] summarized driver mutations in the context of BC subtypes. Here, we focus on the ER mutations and present main driver mutations; in particular, breast cancer subtypes (Figure 3). The *ESR1* gene encodes the estrogen receptor alpha (ERα), which is a key regulator of numerous biological processes, including cell growth and division. In preclinical and clinical studies, it has been observed that *ESR1* mutations appear in the early stages of breast cancer development. Furthermore, as the cancer disease progresses, their occurrence becomes increasingly prevalent in tumor cells [196]. *ESR1* mutations’ frequency and location is also presented in Figure 4.

*ESR1* mutations were discovered in breast cancer over 30 years ago [198]. However, their significant role in endocrine therapy resistance was only proven in 2013 via the sequencing of the metastatic breast cancer (MBC) genome [199]. These studies have demonstrated that *ESR1* mutations are more common in metastatic breast cancer than in primary tumors and may contribute to hormonal therapy resistance. However, *ESR1* mutations alone only partially account for hormonal therapy resistance in MBC. For instance, approximately 50% of hormonal resistance cases are linked to the *ESR1* mutation. Other increasingly identified mechanisms include alterations in the PI3K-AKT-mTORC1, RAS-MAPK, and CDK4/6-RB-E2F signaling pathways, as well as the loss, amplification, and translocation of *ESR1*. Moreover, the *ESR1* mutations usually co-occur with other genomic changes, resulting in an overall worse prognosis [200]. There are several methods used for detecting *ESR1* mutations. Mutations are detected in tumor cells (tumor biopsy), circulating tumor cells (CTCs), and extracellular DNA (cfDNA—cell-free DNA). Common detection methods include next-generation sequencing (NGS) and droplet digital PCR (ddPCR) [201]. All mutations associated with the resistance of ESRα are located in the ligand-binding domain (LBD). The most commonly encountered ones are D538G and Y537S, while less common ones include Y537N, Y537C, L536H, L536P, L536R, S463P, and E380Q [202]. Mutations Y537S and D538G occur in the N-terminal portion of helix 12 (H12) in the ERα domain, which is responsible for ligand binding [203]. The Y537S mutation in the *ESR1* gene leads to the substitution of serine for tyrosine at position 537 in the gene. In the case of the Y537S mutation, it has been observed that the amino acid serine at position 537 forms a hydrogen bond with asparagine at position 351 of the ERα protein. This interaction leads to a change in the structure of the loop between helices 11 and 12; thus, it could potentially contribute to the sustained activity of the protein carrying the Y537S mutation. It has also been discovered that the surface mutation Y537S does not affect the structure of the ligand-binding domain, which aligns with the functional research results showing that the protein remains sensitive to the action of antiestrogens [204]. The Y537S mutation in the *ESR1* gene may also lead to the situation in which cancer cells start to migrate, contributing to distant metastases. To comprehend the pathomechanism associated with this mutation, experimental models were used. These models involved genetic modifications using CRISPR-Cas9 technology to include the Y537S mutation in the *ESR1* gene [205,206]. Transcriptional profiling has revealed that introducing this mutation into the *ESR1* gene increases the activity of the certain signaling pathways characteristic of tumor development, including p53 and the MTORC1 pathway. This suggests that mutated estrogen receptors may play a crucial role in promoting a tumor phenotype that is resistant to endocrine therapy (ET) and prone to metastasis [207]. In the case of the wild-type (ESR1-WT) without mutations, activation occurs through binding to the estrogen ligand. However, *ESR1* with a mutation (ESR1-MUT) in the ligand-binding domain displays constitutive receptor activity and is independent of the presence or absence of the ligand. As a result, treatments based on receptor inhibition or inhibiting the synthesis of its ligands (estrogens) prove to be less effective, since the mutated receptor remains active regardless of ligand availability [203]. Under conditions where the ligand is absent, ESR1-MUT exhibits greater stability in its active conformation, increased the binding to co-activators, and reduced the proteolytic degradation compared to ESR1-WT [208,209]. At the molecular level, ESR1-MUT transactivates the altered sets of target genes, leading to increased cell motility and likely promoting metastasis formation [210]. It has been demonstrated that ESR1-MUT exhibits slightly altered interactions, such as enhanced binding with FOXA1 and GREB1 [211]. Furthermore, even in the presence of estrogen, ESR1-MUT can exhibit a significantly higher transactivation capacity compared to ESR1-WT [199]. Conformational changes in ESR1-MUT lead to the reduced binding of inhibitors, increased coactivator recruitment, and enhanced proteolytic stability, affecting the resistance to aromatase inhibitors (AIs), tamoxifen, and fulvestrant in vitro. Ultimately, the observation that higher doses of tamoxifen and fulvestrant still exhibit efficacy, along with determining the structure of ESR1-MUT, has contributed to the development of new molecules aimed at inhibiting ESR1-MUT. These molecules could be highly effective in targeted therapy against ER [209].

## 7. Therapeutic Targeting of ERs Pathways for Metastatic Control

It is widely known that the main reason for resistance to endocrine therapy (ET) is the complexity of the regulation of estrogen signaling in combination with crosstalk to the other oncogenic signaling pathways [212,213,214]. In BC therapy, targeting the ER signaling pathways plays a pivotal role [149]. However, the molecular mechanisms of these pathways in BC are still unrevealed. Therefore, it is necessary to understand the versatility of molecular mechanisms in different BC types and their divergent signaling. Here, we summarize numerous reports on the therapeutic targeting of ERα signaling for blocking BC metastasis, with a focus on the latest and most promising therapies. Upon the discovery of the ERα (*ESR1*) and ERβ (*ESR2*) receptors’ implication in BC, targeted therapies against these receptors became the center of scientific interest. Targeting the ERα, ERβ, and GPER signaling components involved in enhancing the cell migration, invasion, and EMT processes have also been set in the drug discovery pipeline [215].

**Figure 5:**
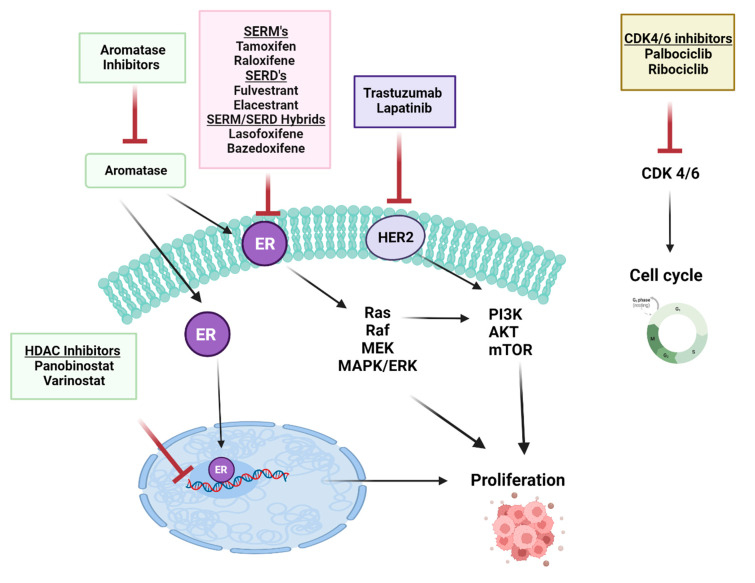
The schematic diagram of the selected main drugs used in breast cancer therapy. Created with BioRender.com.

There are several ways to effectively regulate metastasis in breast cancer patients by targeting the ERα pathway. A schematic diagram of main drugs used in BC treatment is presented in Figure 5. Two main classes of endocrine therapy exist: aromatase inhibitors, (AIs) such as letrozole, anastrozole (non-steroidal AI), and exemestane (steroidal AI), and antiestrogens: ER modulators (SERMs), their function is to block the activation of ERα in BC, as exemplified by tamoxifen (TAM, Nolvadex, Astra Zeneca, Cambridge, UK) [216], chlorinated derivative toremifene [217,218], and raloxifene [219]; and the selective ER downregulators (SERDs), which bind to ERα and leads to a reduced ERα level and activity due to its decay; for example, fulvestrant [220]. These compounds inhibit breast cancer progression by interfering with ER signaling [221]. Currently, the mechanism combining the activities of both SERMs and SERDs has gained much attention. Mixed SERM/SERDs (the so-called SERM/SERD hybrids) such as lasofoxifene (laso) [222] and bazedoxifene [223,224] have been proposed as a potential treatment of ER-positive metastatic BC. These SERM/SERD dual compounds in breast cancer have recently been reviewed in [225,226].

In metastatic carcinoma, it has been found that the ERα and aromatase levels are higher than in normal tissue [227]. Based on the addiction to estrogen signaling by ERα-positive tumors, therapies inhibiting the ERα directly, e.g., by using estrogen antagonists like tamoxifen, or indirectly, e.g., blocking estrogens using aromatase inhibitors, are the principal treatment for ER+, ER-, PR+, or HER2+ BC carriers [228].

Recent studies have found that a series of genetic and epigenetic modifications play a role in resistance mechanisms; thus, these can be used as alternative targets in ER+ breast cancer. As described above, the ERα crosstalks with the other signaling pathways, including growth factor receptor signaling such as HER family elements, the fibroblast growth factor receptor (FGFR) pathways, intracellular growth, and the metabolic pathway with survival signals PI3K/Akt/mTOR. ERα also interacts with HDACs, by which ERα can regulate gene expression, along with CDK4 and 6 (cyclin-dependent kinases 4 and 6, which are the main regulators of the cell cycle). Research on the development of the inhibitors of these pathways contributes to the better effectiveness of hormone therapy in the treatment of early and metastatic tumors [229]. Furthermore, phosphatases, proteases, miRNAs, and long non-coding RNAs (lncRNAs) might also be potential therapeutic targets [230].

Targeting the EGFR pathway, hormone therapy in use with HER2-targeted agents is an alternative to current chemotherapy regimens in the fight against metastasis. Combination therapies (hormonal and targeted) have recently been approved and tested, and hormonal drugs are also still being studied as monotherapy [231]. The most widely used therapeutics for breast cancer are the agents that target ERs and HER2, such as TAM and trastuzumab [232]. Another class of therapeutics agents developed is associated with FGFR inhibitors, which are responsible for reverse endocrine resistance in BC. That mechanism is based on the fact that FGFR inhibitors can restrain or reverse acquired multidrug resistance (MDR) by directly blocking the efflux of ATP-binding cassette (ABC) transporter proteins, and they play a pivotal role in overcoming chemotherapy resistance [233]. At present, FGFR inhibitor AZD4547 is studied in combination with AIs such as letrozole or anastrozole in patients with cancer progression [234].

The analysis of the targeting of the PI3K/Akt/mTOR pathway shows that the combined targeting of the ERα and estrogen XPO1 affects metabolic pathways, inhibits Akt activation, and causes autophagy, ultimately reversing TAM resistance [235]. Selinexor (SEL), an XPO1 antagonist, has been used in many clinical trials to overcome hormonal resistance [236,237]. Transcriptomic analysis showed that 4-hydroxy tamoxifen (4-OHT), SEL alone or in combination, induced different gene expression profiles related to Akt signaling and metabolism [235]. The same group of researchers reported the effective combination of TAM and SEL suppressing progression by restraining the growth of metastatic ER-positive tumors in vivo [238]. The GAS5 lncRNA is downregulated in BC. The combination of GAS5 restoration and HER2 inhibition has been shown to have a synergistic effect in inhibiting BC cell proliferation and migration. Also, in trastuzumab-resistant BC cells, lapatinib increased GAS5 by inhibiting the mTOR pathway [239]. miR-221 and miR-222 have also been shown to target the ERα pathway by suppressing the expression of the tumor suppressor gene, *PTEN*. The inhibition of miR-221 and miR-222 has been suggested as a therapeutic strategy [240]. Beyond these, targeting other miRNAs such as miR-17-5p [241], miR-27a [242], and miR-206 [243] might be a novel therapeutic strategy in BC.

The CDK4/6-targeted inhibitor treatment of advanced hormone-resistant and metastatic ER+ breast cancer has shown significant clinical benefit when combined with aromatase inhibitors (AIs) or selective estrogen receptor degraders (SERDs) [244]. The clinical success of CDK4/6 inhibitors led to their approval by the FDA. Palbociclib (Ibrance), ribociclib (Kisqali), and abemaciclib (Verzenio), in combination with letrozole (AI) or fulvestrant (SERDs), have been used as effective initial or subsequent therapies [244,245].

In comparison to AI therapy, fulvestrant (SERD) treatment does not select *ESR1* mutations conferring the ligand-independent and constitutive activation of ERα [246]. The mutated *ESR1* is resistant to estrogen degradation in contrast to the wild-type *ESR1* and is definitely less susceptible to tamoxifen or fulvestrant. To make treatment more efficient, it is necessary to develop a new generation SERM or SERD for breast cancer with the *ESR1* mutation or even new strategies that are successful in targeting the mutated ER. The pursuit of this has led to the development and characterization of the second and third generation hybrids of SERD, SERM, and SERDs-SERMs, which are currently in phase I/II clinical trials [247,248]. Notably, a novel first, orally bioavailable SERD, elacestrant (RAD1901) [249,250], has undergone a phase III clinical trial [251]. This phase III randomized clinical study called EMERALD compared elacestrant with the standard currently used monotherapy with the fulvestrant or aromatase inhibitor in ER-positive/HER-negative metastatic breast cancer. Elacestrant triggered progression-free survival in all patients and in patients who have developed mutations in *ESR1* [251]. Moreover, Patel and colleagues [252] have shown that elacestrant exhibits anti-cancer activity in the cells resistant to all CDK4 and 6 inhibitors approved to date, namely palbociclib, abemaciclib, and ribociclib in both wild-type and mutated *ESR1*. In January 2023, elacestrant (as ORSERDU™, Stemline Therapeutics, Menarini Group, Florence, Italy) received FDA approval for the treatment of adult patients ER-positive/HER2-negative *ESR1*-mutated advanced or metastatic BC (developments summarized recently by [253]). Future research prospects may focus on testing the status of the *ESR1* mutations as a therapeutic target and can be used as therapies associated with clinical benefits for cancer patients [254].

Studying another agent from the epigenetic modifier group, research showed that in most BC, the *ESR1* is methylated on its CpG island, which results in gene repression. By changing the epigenetic landscape using HDAC inhibitors, the *ERS1* gene is derepressed, restoring the sensitivity of the ER to TNBC tumors [255]. Moreover, HDAC inhibitors did not cause any changes alone, while had synergistic effect in combination with, e.g., cisplatin [256]. The same synergistic effects were observed for HDACs use in combination with other treatments, such as aromatase inhibitors or radiation [257,258,259,260]. In addition, synthetic lethality, where a single target has no effect but two targets result in cell death, occurs in TNBC cells with simultaneous treatment when the HDAC inhibitors are combined with the PARP or cisplatin inhibitors [261,262]. One of the HDAC inhibitors being studied in clinical trials for the treatment of TNBC is an entinostat. It has been shown that entinostat induces ERα expression and sensitizes TNBC cells to hormonal therapies such as letrozole [263]. In a phase II clinical trial, entinostat, combined with exemestane, an aromatase inhibitor, revealed promising results in postmenopausal women with ER-positive, HER2-negative metastatic breast cancer [258,264].

## 8. The Summary of the Latest Developments

It is estimated that by 2040, there will be more than 3 million BC cases per year, with more than 1 million deaths [4]. Breast tumors are heterogeneous in nature but most express estrogen receptors. Since ER regulates the transcription of many genes via its genomic and nongenomic actions, it became an important therapeutic target. The assessment of ER expression lays the foundations for the diagnostic workflow of BC patients and serves as a biomarker for the prediction of endocrine therapy [6,265]. Over the past few years, considerable progress has been achieved in the development of drugs targeting BC. Endocrine therapy is a current treatment strategy for both early and advanced stages of ER-positive BC. This therapy targets the ER pathway at different levels and includes different compounds. These are aromatase inhibitors (reduces circulating estrogen, thus acts on the ER stimulus), but also antiestrogens classified as SERMs or SERDs (directly inhibits the ER with the first orally administered SERD elacestrant being approved for BC treatment) [251,253]. However, dual compounds, the so-called SERM/SERD hybrids, have recently gained much attention [222,223]. Depending on the cancer type and the disease advancement, either monotherapy (one compound) or combination therapy (more than one compound) is used for the treatment. The mutations in the ER-coding gene, the cross-talk between various receptors, and the ER post-translational modifications make the treatment more challenging, but also opens the door for other promising compound developments, especially to overcome the problem of acquired resistance of the ER-positive BC [266].

## 9. Conclusions

Estrogen receptors orchestrate many cellular functions. Abnormal ER signaling may result in various cancers, including breast cancer, a second cause of female cancer-related death. Cancer is a multifactorial, complex disease; however, the majority of breast tumors express ER. The critical function of ERs in the growth and survival of hormone-dependent cancer cells makes them important targets for both diagnostic and therapeutic purposes. ER-signaling involves either genomic nuclear mechanisms or non-genomic intracellular cascades for transcriptional control. The mechanisms of action of ER are even more versatile with its coregulators (coactivators and corepressors), with many protein–protein interactions and post-translational modifications involved. The mutations of ER-encoding genes appear already at the early stages of BC and are more prevalent once the disease progresses. Given the complexity of ER signaling in breast cancer, there is a need to unravel the molecular and cellular mechanisms that modulate ER signaling in BC. Thus, novel combination treatment strategies and new molecules that also target the post-translational mechanisms should be investigated.

## Figures and Tables

**Table 1:** Nuclear estrogen receptors’ coregulators.

**Coactivators**	**Influence on ERα**
AIB1	The AIB1 coactivator is one of the transcription factors that react with ERα in a ligand-dependent manner, and its coactivator activity is enhanced by the CIB1δ and PKCε-mediated phosphorylation of AIB1. This action results in an increased expression of target genes, e.g., those responsible for cell migration, such as *PEA3* (the polyomavirus enhancer activator 3), *MMP2* (metalloproteinase 2), and *MMP9* (metalloproteinase 9), and it is therefore directly related to tumorigenesis and metastasis [107,108]. The AIB1 coactivator activates ERα-dependent transcription by recruiting HAT to the chromatin of the *ESR1* gene. In addition, the AIB1 protein is involved in the regulation of the degradation of the ERα via the ubiquitin–proteasome system (UPS) [109].
BCAS3	The highly conserved BCAS3 (breast cancer-amplified sequence 3) coactivator, like AIB1, interacts with ERα’s transcriptional complex, in conjunction with PELP1’s (proline-, glutamic acid-, and leucine-rich protein 1) coactivator, causing the activation of ERα-encoding gene transcription [110].
DBC1	It has been demonstrated that the DBC1 (deleted in breast cancer 1) protein, a negative regulator of deacetylase SIRT1, functions as a nER coactivator, and it is essential for the formation of the ER transcription complex and the proliferation of estrogen-dependent breast cancer cells. The deletion of DBC1 from ER-negative breast cancer cells was shown to decrease cell proliferation in vivo and in vitro, and increased DBC1 expression resulted in a negative prognosis and shortened recurrence-free survival in the ER-negative patients [111]. In addition, DBC1 overexpression is observed in prostate, gastric, esophageal, and colorectal cancers and has led to a worsening of the predicted poor prognosis [112].
PELP1	PELP1 regulates the genomic and non-genomic ERα signaling. It interacts with many transcription factors, and its activity is observed in the cell nucleus, cytosol, and plasma membrane [113,114]. It has an important role in the remodeling of chromatin by interacting with histones and histone-modifying enzymes [115]. PELP1 causes the activation of tyrosine kinase SRC, resulting in the reorganization of the cell cytoskeleton [116,117]. Increased PELP1 expression has been observed as a result of enhanced tumor cell invasion [118]. The effect of PELP1 is an epigenetic modification, leading to ERα activation [113]. PELP1 has been proposed as a biomarker of hormone-dependent cancers, i.e., ovarian and breast cancer [115,119].
CIZ1	CIZ1 (Cip1-interacting zinc-finger protein), a DNA-binding protein, is implicated (as an ER coactivator) in the ER transactivation due to the cooperation of the ER to the chromatin target gene. In addition, the overexpression of CIZ1 causes an increase in sensitivity to estrogen, accelerating the growth rate of breast cancer cells [120]. The increased expression of CIZ1 is observable not only in breast cancer but also in cancers like colon, lung, gallbladder, prostate, and other diseases, e.g., rheumatoid arthritis [121].
**Corepressors**	**Influence on ERα**
NCOR1	NCOR1 (nuclear receptor corepressor 1) inhibits ERα expression, suppressing transcription through the ligand-binding domain of ERα [122]. NCOR1 regulates the availability of chromatin by activating histone deacetylase 3 (HDAC3) [123,124,125]. In addition, it acts antagonistic on histone acetyltransferase (HAT) and the HAT-activating enzyme, causing the inhibition of its expression, which promotes the formation of compact, inactive heterochromatin [126]. The loss of NCOR1 results in accelerating the development of breast cancer, and a decrease in its expression may be the result of acquiring resistance to tamoxifen [127,128]. Additionally, it has been shown that the association of NCOR1 with other corepressors such as SAFB1 (scaffold attachment factor B 1) and SAFB2 (scaffold attachment factor B 2) reduces the expression of ERα [129,130]. Recently, Aylon and colleagues reported [131] that NCOR1 repressive activity is enhanced by LAST1 (large tumor suppressor 1) and proposed that this axis may restrict breast cancer progression.
BRCA1	BRCA1 is the corepressor of ERα that works by binding to the AF-2 ERα domain, thanks to which it leads to the monoubiquitylation of the ER together with BARD1 influencing ER activity [132]. In non-immortalized fibroblasts and breast cancer cells, BRCA1 deficiency has been shown to activate the PI3K/AKT pathway by accumulating AKT. This effect is reinforced by the fact that estrogen also activates the PI3K/AKT pathway in the ER-dependent and independent manner. Therefore, it has been shown that in the BRCA1-deficient breast cancer cells, estrogen causes the initiation of the carcinogenesis process by stimulating cell division via the AKT pathway and activating the epithelial–mesenchymal transition (EMT) [133].
DACH1	DACH1 (Dachshund 1) is one of the ER corepressors, which works by blocking its action [134]. It regulates gene expression by binding to DNA-binding transcription factors and by blocking DNA strands [135]. The downregulation of the transcription of MMP9 by DACH1 inhibits breast cancer tumor cells’ invasion and metastasis [136]. DACH1 also inhibits the growth of cancer stem cells (CSCs), resulting in the inhibition of metastasis [137,138]. Moreover, it was shown that DACH1 suppresses breast cancer via a negative regulation of CD44 (cluster of differentiation-44) [139]. DACH1 interacts with the ER by blocking the interaction between *ESR1* and the activator, resulting in an increased activity of HDAC and reduced ER transcription [134]. DACH1 expression is upregulated in individuals who show longer disease-free survival, ER-positive breast cancer-free survival, and reduced metastasis [140].
